# Comparative characterisation of human and ovine non-*aureus* staphylococci isolated in Sardinia (Italy) for antimicrobial susceptibility profiles and resistance genes

**DOI:** 10.1017/S0950268821000212

**Published:** 2021-01-29

**Authors:** C. M. Longheu, E. Azara, S. Attene, S. Sanna, M. Sale, M. F. Addis, S. Tola

**Affiliations:** 1 Istituto Zooprofilattico Sperimentale della Sardegna ‘G. Pegreffi’, 07100 Sassari, Italy; 2 Ospedale ‘San Francesco’, 08100 Nuoro, Italy; 3 Azienda Ospedaliera Universitaria, 07100 Sassari, Italy; 4 Ospedale ‘A. Segni’, 07014 Ozieri, Italy; 5 Dipartimento di Medicina Veterinaria, Università degli Studi di Milano, 20133 Milano, Italy

**Keywords:** Human, MLST, non-*aureus* staphylococci, ovine, PCR-RFLP, resistance genes

## Abstract

We present the comparative characterisation of 195 non-*aureus* staphylococci (NAS) isolates obtained from sheep (*n* = 125) and humans (*n* = 70) in Sardinia, Italy, identified at the species level by *gap* gene polymerase chain reaction (PCR) followed by restriction fragment length polymorphism analysis with *Alu*I. Isolates were tested phenotypically with a disc diffusion method and genotypically by PCR, for resistance to 11 antimicrobial agents including cationic antiseptic agents. Among the ovine isolates, *Staphylococcus epidermidis* (*n* = 57), *S. chromogenes* (*n* = 29), *S. haemolyticus* (*n* = 17), *S. simulans* (*n* = 8) and *S. caprae* (*n* = 6) were the most prevalent species, while among human isolates, *S. haemolyticus* (*n* = 28) and *S. epidermidis* (*n* = 26) were predominant, followed by *S. lugdunensis* and *S. hominis* (*n* = 4). Of the 125 ovine isolates, 79 (63.2%) did not carry any of the resistance genes tested, while the remainder carried resistance genes for at least one antibiotic. The highest resistance rates among ovine isolates were recorded against tetracycline (20.8%), and penicillin (15.2%); none was resistant to methicillin and two exhibited multidrug resistance (MDR); one of which was positive for the antiseptic resistance *smr* gene. By contrast, most human isolates (59/70, 84.3%) were resistant to ⩾1 antimicrobials, and 41 (58.6%) were MDR. All 52 (74.3%) penicillin-resistant isolates possessed the *bla*Z gene, and 33 of 70 (47.1%) harboured the *mec* gene; of these, seven were characterised by the Staphylococcal Chromosomal Cassette (SCC*mec*) type IV, 6 the type V, 5 of type III and one representative each of type I and type II. The majority (57.1%) was erythromycin-resistant and 17 isolates carried only the efflux *msr*A gene, 11 the methylase *erm*C gene and an equal number harboured both of the latter genes. Moreover, 23 (32.8%) were tetracycline-resistant and all but one possessed only the efflux *tet*K gene. *qac*A/B and *smr* genes were detected in 27 (38.6%) and 18 (25.7%) human NAS, respectively. These results underline a marked difference in species distribution and antimicrobial resistance between ovine and human-derived NAS.

## Introduction

Sardinia, an island located in the middle of the Mediterranean Sea, with a population is around 1.6 million inhabitants, has approximately 3.5 million milking Sarda sheep, corresponding to half of the total Italian national stock. A considerable part of the regional economy relies on dairy sheep farming, mainly for pecorino cheese production; as a consequence, the control of intra-mammary infections is of the greatest importance for dairy farmers. Several reports indicate that non-*aureus* staphylococci (NAS) are the most prevalent bacteria recovered from subclinical mastitis of sheep and goats [1–4], thus creating opportunities for cross colonisation and infection among sheep and farmers, due to their antimicrobial resistance and pathogenicity gene pools. Of note, NAS have emerged as relatively frequent nosocomial agents capable of causing infection in debilitated or compromised patients as well as their association with catheter-related and other indwelling medical device-related infections [5].

In the last decade, a significant increase of antibiotic-resistant infections has been recorded among ovine NAS, especially for beta-lactams and tetracyclines, which are commonly used in veterinary practice for mastitis treatment [6, 7]. Two mechanisms confer penicillin resistance in staphylococci, the most common being production of β-lactamase, encoded by the *bla*Z gene. The other mechanism is due to a penicillin-binding protein transpeptidase (PBP2a), encoded by the *mecA* gene [8], which is carried on a mobile chromosomal element, the Staphylococcal Chromosomal Cassette *mec* (SCC*mec*) [9]. SCC*mec* types are defined by the recombinase (*ccr*) gene complex and the class of the *mec* gene complex [10]. Recently, a novel PBP2a homologue has been described as encoded by *mec*C [11].

Many cationic antiseptic agents such as quaternary ammonium compounds (QACs) are widely used as surface germicides within healthcare facilities [12, 13]. Although issues of antibiotic resistance have been widely investigated, knowledge on the occurrence of antiseptic resistance genes (*qac*A/B, *smr*, *qac*G, *qac*H, *qac*J) in staphylococci from dairy animals is limited [7, 14].

In most veterinary and clinical laboratories, differentiation of NAS species is based on phenotypic reactions which may be unreliable, particularly for animal isolates. Consequently, several genotypic methods are increasingly being applied for species-level identification [15]. This approach combined with genotyping by multilocus sequence typing (MLST) for the differentiation of strain populations, allows an informative analysis and insight into the evolving epidemiology of bacterial species groups in relation to their pathogenicity and antimicrobial resistance [16].

The objective of this study was to compare the molecular characteristics of NAS isolated from ovine mastitis with those from human clinical specimens, and specifically to: (1) identify NAS isolates using genotypic techniques; (2) determine their antimicrobial susceptibility profiles, and distribution of antimicrobial and antiseptic resistance genes and (3) determine the genetic relatedness of isolates within species by MLST.

## Materials and methods

### Isolate collection

#### Ovine

In total, 125 NAS isolates were collected from sheep milk samples in different provinces of Sardinia (Italy) over a period of 9 months (April–December 2017). The isolates belonged to a bank of NAS used for the preparation of inactivated autogenous vaccines, according to the Italian Ministerial Decree no. 287/1994. Basic identification of staphylococci was determined by colony morphology, Gram-stain, catalase and coagulase tests, clumping factor production (Staphylase Test, Oxoid, UK), and growth on mannitol salt agar (Becton Dickinson, Quebec, CDN).

#### Human

During the same period, 70 NAS isolates were collected from clinical specimens from 70 different patients attending clinical departments at three major hospitals in Sardinia. Isolates were anonymised without patient identifiers and thus individual consent was not required: 90% of the human NAS were recovered from hospitalised patients in intensive care unit, haematology and orthopaedics.

### Species identification by polymerase chain reaction-restriction fragment length polymorphism (PCR-RFLP)

Species identification was based on PCR amplification and PCR-RFLP of the glyceraldehyde-3-phosphate dehydrogenase gene (*gap*) [17]. Sequencing of the *gap* gene was used to identify non-speciated isolates. Briefly, 15 μl of amplicons were digested in a 30 μl volume containing 10× buffer, 0.5 μl of 10 mg/ml acetylated BSA (Promega, Madison, USA) and 1 μl of 10 U/μl FastDigest *Alu*I endonuclease (Thermo Fisher Scientific, City, Country). Samples were incubated at 37 °C for 15 min and then electrophoresed in 12% polyacrylamide gel. Fifteen reference strains were included: *Staphylococcus epidermidis* ATCC 14990, *S. chromogenes* ATCC 43764, *S. xylosus* ATCC 29971, *S. warneri* ATCC 27836, *S. simulans* ATCC 27848, *S. capitis* ATCC 27840, *S. hominis* ATCC 27844, *S. haemolyticus* ATCC 29970, *S. arlettae* ATCC 43957, *S. caprae* ATCC 35538, *S. saprophyticus* ATCC 15305, *S. equorum* ATCC 43958, *S. lentus* ATCC 29070, *S. hyicus* ATCC 11249 and *S. aureus* ATCC 43300.

### Antimicrobial susceptibility testing

Antimicrobial susceptibility testing was performed using a disc diffusion method on Mueller–Hinton agar plates conforming to the Clinical and Laboratory Standards Institute [18] with an inoculum corresponding to the 0.5 McFarland standard. The following antibiotic discs (Oxoid, Basingstoke, UK) were used: penicillin (Pn, 10 IU), tetracycline (Te, 30 μg), streptomycin (St, 10 μg), kanamycin (Km, 30 μg), gentamicin (Gn, 10 μg), erythromycin (Er, 15 μg), trimethoprim-sulphamethoxazole (Trs, 25 μg), amoxicillin-clavulanic acid (Amc, 30 μg), cephalothin (Kf, 30 μg), cefoxitin (Kx, 30 μg, for detection of methicillin resistance) and oxacillin (Ox, 1 μg). *S. aureus* ATCC 25923 were used as the quality control strain. Isolates were classified as susceptible, intermediate or resistant based on inhibition zone diameters using CLSI breakpoint values (mm) for *S. aureus*. Multidrug resistance (MDR) was defined as resistance to at least three classes of the antimicrobials tested [19].

### Detection of resistance genes and SCC*mec* typing

Cefoxitin/oxacillin-resistant isolates were tested for carriage of *mec*A gene and SCC*mec* type by PCR [20]. The presence of *ccr*AB5 and of the novel *ccr* allotype (*ccr*AB_SHP_) was confirmed with published primer sets [21, 22]. Genes encoding resistance to penicillin (*bla*Z), macrolide (*msr*A, *erm*A, *erm*B and *erm*C) and tetracycline (*tet*O, *tet*K, *tet*M and *tet*L) were also identified by PCR [23–26]. The following positive controls were included: *S. aureus* ATCC 33591 (*bla*Z), *S. haemolyticus* isolate 772 (*erm*A and *msr*A), *Enterococcus faecalis* 8855 (*erm*B), *S. haemolyticus* 15680 (*erm*C), *S. aureus* 4438 (*tet*K), *S. epidermidis* 1464 (*tet*L), *S. aureus* 2412 (*tet*M) and *S. aureus* 4438 (*tet*O).

Genes encoding resistance to antiseptics (*qacA*/*B* and *smr*) were screened for by a multiplex PCR using published primer sets [27]. *S. haemolyticus* isolates 11628 and 8869 were used as positive controls for *qacA*/*B* and *smr* genes, respectively.

#### MLST

Amplification of the seven housekeeping genes (*arc*C, *aro*E, *gtr*, *mut*S, *pyr*R, *tpi*A and *yqi*L) was performed using the primer sequences as previously described [28]. Gene fragments were amplified by conventional PCR (GeneAmp PCR System 9700, Applied Biosystems, Foster City, CA, USA), and allele sequences were assigned and compared with reference to the *S. epidermidis* MLST database (https://pubmlst.org/sepidermidis/).

## Results

### Ovine NAS

**Fig. 1.**
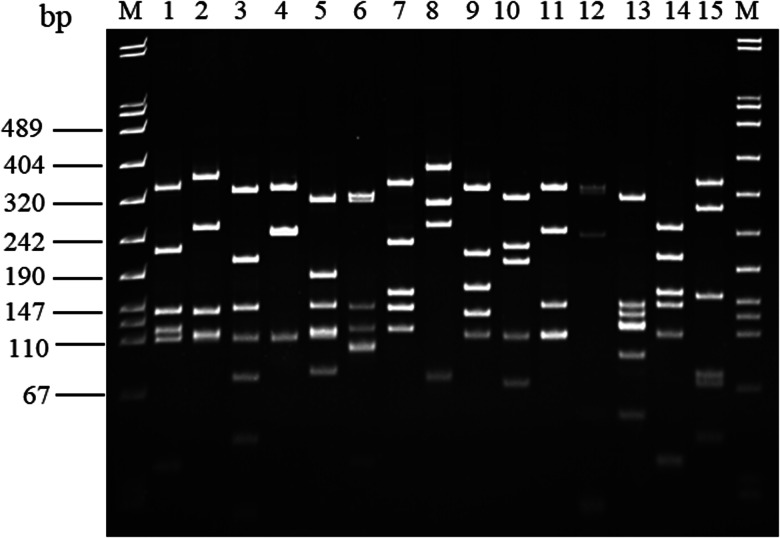
RFLP patterns obtained for 15 reference strains after digestion of the *gap* gene amplicon with *Alu*I. Fragments were separated by 12% PAGE gel. Lane M, marker VIII (Roche); lane 1, *S. xylosus* ATCC 29971; lane 2, *S. saprophyticus* ATCC 15305; lane 3, *S. capitis* ATCC 27840; lane 4, *S. haemolyticus* ATCC 29970; lane 5, *S. simulans* ATCC 27848, lane 6, *S. warneri* ATCC 27836; lane 7, *S. arlettae* ATCC 43957; lane 8, *S. chromogenes* ATCC 43764; lane 9, *S. equorum* ATCC 43958; lane 10, *S. hominis* ATCC 27844; lane 11, *S. caprae* ATCC 35538; lane 12, *S. lentus* ATCC 29070; lane 13, *S. hyicus* ATCC 11249, lane 14, *S. epidermidis* ATCC 14990 and lane 14, *S. aureus* ATCC 43300.

Figure 1 shows the PCR-RFLP profiles obtained for the 15 reference *Staphylococcus* strains. All, but two, of the 125 ovine isolates were assigned to seven species: *S. epidermidis* (*n* = 57), *S. chromogenes* (*n* = 29), *S. haemolyticus* (*n* = 17), *S. simulans* (*n* = 8), *S. caprae* (*n* = 6), *S. warneri* (*n* = 5) and *S. saprophyticus* (*n* = 1); the remaining two isolates were identified as *S. intermedius* and *S. muscae* (Table 1). Seventy-nine (63.2%) were susceptible to all antibiotics tested and the remaining 46 (36.8%) showed resistance to at least one antibiotic. The highest resistance rate was recorded against Te (*n* = 26; 20.8%), followed by Pn (*n* = 19; 15.2%), St (5.6%) and Er (4%). No resistance to Gn, Amc, Kf, Kx and Ox was detected. Only two isolates (1.6%) showed MDR. No isolate was positive for *qac*A/B genes but one harboured the *smr* antiseptic resistance gene.

**Table 1.** Distribution of ovine and human NAS isolates, identified by PCR-RFLP and sequencing analysis of the *gap* gene, according to the specimen origin

Source	*S. epi*	*S. chr*	*S. hae*	*S. sim*	*S. cpr*	*S. war*	*S. sap*	*S. int*	*S. mus*
*Ovine isolates*^a^									
Milk	**57**	**29**	**17**	**8**	**6**	**5**	**1**	**1**	**1**
	
Source	*S. hae*	*S. epi*	*S. lug*	*S. hom*	*S. cap*	*S. war*	*S. xyl*	*S. pas*	*S. sap*
*Human isolates*^a^									
Nasal swab	6	5	–	–	–	–	–	–	–
Blood	4	6	–	1	–	–	–	–	–
Skin swab	2	3	–	–	2	–	1	–	–
Pus	1	3	–	–	–	–	–	1	–
Peritoneal fluid	2	–	2	–	–	–	–	–	–
Seminal fluid	4	–	–	–	–	–	–	–	–
Injury	–	3	1	–	–	–	–	–	–
Ear swab	2	–	–	–	–	1	–	–	–
Oral swab	1	1	–	–	–	–	–	–	1
Urine	2	–	–	1	–	–	–	–	–
C.V.C^b^	–	2	–	–	–	–	–	–	–
Ulcer swab	–	1	–	–	–	–	–	–	–
F.V.C.^b^	–	1	–	–	–	–	–	–	–
Peritoneal swab	1	–	–	–	–	–	–	–	–
Vaginal swab	–	–	1	–	–	–	–	–	–
Glans swab	1	–	–	–	–	–	–	–	–
Pleural fluid	–	–	–	1	–	–	–	–	–
Fluid drainage	–	–	–	–	–	1	–	–	–
B.L. fluid^b^	1	–	–	–	–	–	–	–	–
N.P. aspirate^b^	–	–	–	–	1	–	–	–	–
Bile	1	–	–	–	–	–	–	–	–
Biopsy	–	1	–	–	–	–	–	–	–
Prosthesis	–	–	–	1	–	–	–	–	–
Total	**28**	**26**	**4**	**4**	**3**	**2**	**1**	**1**	**1**

a Isolate abbreviations: *S. hae*, *S. haemolyticus*; *S. epi*, *S. epidermidis*; *S. chr*, *S. chromogenes*; *S. sim*, *S. simulans*; *S. lug*, *S. lugdunensis*; *S. hom*, *S. hominis*; *S. cpr*, *S. caprae*; *S. cap*, *S. capitis*; *S. war*, *S. warneri*; *S. xyl*, *S. xylosus*; *S. pas*, *S. pasteuri*; *S. int*, *S. intermedius*; *S. sap*, *S. saprophyticus* subsp. *bovis*; *S. mus*, *S. muscae*.

b Specimen abbreviations: C.V.C., central venous catheter, F.V.C., femoral venous catheter, B.L. fluid, bronchoalveolar lavage fluid, N.P. aspirate, nasopharyngeal aspirate.

#### S. epidermidis

*S. epidermidis* was the most represented species among ovine isolates. Of the 57 isolates, 22 (38.6%) were resistant to one or more antimicrobials with Te- and Pn-resistance in 26.3% and 14% of all isolates, respectively. Two were MDR: one was resistant to three different classes (Pn, Er and Te) and one to four different classes (Pn, Te, Km and Trs) (Table 2). All Pn-resistant *S. epidermidis* had the *bla*Z gene while all Te-resistant isolates harboured only *tet*K. The Er-resistant isolate carried *msr*A, and the isolate resistant to four different antibiotics was positive for *smr* (Table 2).

**Table 2.** Antimicrobial susceptibility results, resistance genes detected and SCC*mec* typing of both 57 ovine and 26 human *S. epidermidis* isolates

Source	Antimicrobial resistance^a^	Resistance genes^b^	SCC*mec* C*mec* type
*Ovine isolates*			
Milk	Pn (*n* = 4)	*blaZ*	–
	Te (*n* = 10)	*tetK*	–
	Km (*n* = 1)	–	
	St (*n* = 1)	–	
	Pn-Te (*n* = 1)	*blaZ- tetK*	–
	Pn-St (*n* = 1)	*blaZ*	–
	Te-St (*n* = 2)	*tetK*	–
	Pn-Te-Er (*n* = 1)	*blaZ- tetK-msrA*	–
	Pn-Te-Km-Trs (*n* = 1)	*blaZ- tetK-smr*	–
*Human isolates*			
Pus	–	*smr*	–
Blood	Pn	*blaZ*, *smr*	–
Biopsy	Pn	*blaZ*	
Skin swab	Er	*msr*A, *qac*A/B	–
Ulcer swab	Gn-Km	*qac*A/B	–
Oral swab	Gn-Km	–	–
Pus	Pn-Te	*blaZ*, *tet*K,	–
Nasal swab	Pn-Ox	*blaZ*, *smr*	NT
Nasal swab	Pn-Er-Ox	*blaZ*, *erm*C,	Type IV
C.V.C.^c^	Pn-Er-Ox	*blaZ*, *erm*C, *msr*A, *smr*	Type III
Injury	Pn-Te-Ox	*blaZ*, *tet*K, *qac*A/B	Type IV
Skin swab	Pn-Er-Trs	*blaZ*, *erm*C	–
Blood	Pn-Km-Ox	*blaZ*, *smr*	Type IV
Injury	Pn-Er-Gn-Km	*blaZ*, *msr*A, *qac*A/B	–
Blood	Er-Gn-Km-Trs	*erm*C, *msr*A, *qac*A/B	–
Skin swab	Pn-Gn-Km-Ox	*blaZ*,	NT
Blood	Pn-Er-Gn-Km	*blaZ*, *msr*A, *qac*A/B	–
Nasal swab	Pn-Er-Gn-Km-Ox	*blaZ*, *msr*A, *smr*	Type IV
F.V.C.^c^	Pn-Er-Gn-Km-Ox	*blaZ*, *erm*C	NT
Pus	Pn-Gn-Km-Amc-Ox	*blaZ*, *qac*A/B	NT
Nasal swab	Pn-Er-Te-Amc-Ox	*blaZ*, *erm*C, *tet*K, *tet*L, *smr*	Type II
Injury	Pn-Er-Gn-Km-Te-Trs	*blaZ*, *erm*C, *msr*A, *tet*K, *smr*	–
Blood	Pn-Er-Gn-Km-Trs-Ox	*blaZ*, *msr*A, *qac*A/B	Type III
Nasal swab	Pn-Er-Gn-Km-Amc-Trs-Ox	*blaZ, msr*A, *qac*A/B	Type III
C.V.C.^c^	Pn-Er-Gn-Km-Amc-Trs-Ox	*blaZ*, *erm*C, *smr*	Type III
Blood	Pn-Er-Gn-Km-Amc-Trs-Ox	*blaZ*, *erm*C	Type III

a Antibiotic abbreviations: Pn, penicillin; Er, erythromycin; Te, tetracycline; Km, kanamycin; Ox, oxacillin, Trs, trimethoprim-sulphamethoxazole; Gn, gentamicin; Amc, amoxicillin-clavulanic acid, St, streptomycin.

b Resistance genes for Pn (*bla*Z), Er (*erm*A, *erm*B, *erm*C, *msr*A), Te (*tet*K, *tet*O, *tet*L, *tet*M), antiseptic agents (*qacA*/*B*, *smr*).

NT, isolate non typeable for SCC*mec*.

c Specimen abbreviations: C.V.C., central venous catheter, F.V.C., femoral venous catheter.

An MLST profile was assigned to 54 of the 57 isolates with the most prevalent being ST225 (*n* = 15), ST6 (*n* = 11) and ST100 (*n* = 5) (Table 3). Eight novel allelic profiles were submitted and designated as ST971, ST974, ST975, ST976, ST977, ST978, ST979 and ST980 in the MLST database (http://sepidermidis.mlst.net/). The two MDR isolates were ST81 and ST979, respectively.

**Table 3.** Distribution of sequence types (ST), allelic profiles (MLST) and source of the 57 ovine and 26 human *S. epidermidis* isolates

ST	MLST profile (*arc*C, *aro*E, *gtr*, *mut*S, *pyr*R, *tpi*A, *yqi*L)	Source (*n*)
225	1-13-7-2-2-1-29	Milk (15)
6	1-1-2-2-2-1-1	Milk (11)
100	2-1-1-2-20-1-1	Milk (5)
9	1-2-2-2-2-1-1	Milk (3)
528	1-1-7-2-2-1-29	Milk (3)
5	1-1-1-2-2-1-1	Milk (1)
81	2-17-1-1-2-1-1	Milk (1)
189	1-1-2-2-2-16-1	Milk (1)
413	1-6-2-2-2-1-1	Milk (1)
971	2-2-1-2-20-1-1	Milk (2)
974	2-1-6-2-1-1-1	Milk (1)
975	1-13-7-2-2-1-6	Milk (3)
976	1-1-7-2-2-1-6	Milk (1)
977	1-13-1-2-2-1-1	Milk (1)
978	1-13-2-2-2-1-1	Milk (1)
979	16-13-2-2-2-1-1	Milk (1)
980	1-13-7-27-2-1-6	Milk (1)
Untypeable		Milk (5)
59	2-1-1-1-2-1-1	Injury (3), pus (2), skin swab (1)
5	1-1-1-2-2-1-1	Nasal swab (3), blood (2), biopsy (1)
80	21-1-2-2-4-1-1	Blood (2), Nasal swab (1), F.V.C.^a^ (1)
7	1-1-1-2-4-1-1	Skin swab (1)
166	1-1-2-2-2-1-3	Skin swab (1)
171	25-19-17-4-9-10-2	Pus (1)
2	7-1-2-2-4-1-1	C.V.C.^a^ (1)
173	1-6-6-1-2-1-10	Blood (1)
35	2-1-2-2-4-1-1	C.V.C.^a^ (1)
60	1-1-2-6-2-1-16	Oral swab (1)
172	1-2-1-2-2-1-1	Skin swab (1)
981	1-1-1-27-2-1-1	Blood (1)
Untypeable		Ulcer swab (1)

a Specimen abbreviations: F.V.C., femoral venous catheter, C.V.C., central venous catheter.

#### *S. chromogenes*, *S. haemolyticus* and minor ovine NAS

*S. chromogenes* (*n* = 29) and *S. haemolyticus* (*n* = 17) were the next most prevalent species isolated from ovine milk samples. Only one of the 17 *S. haemolyticus* isolates was resistant to Te (*tet*K gene) (Table 4). Among *S. chromogenes*, 11 isolates were resistant to a single antibiotic and four to two agents. The highest resistance was found against Pn (*n* = 7), followed by Te (*n* = 6) and Er (*n* = 3). All Pn-resistant isolates had the *bla*Z gene while, among the six *S. chromogenes* isolates resistant to Te, five were positive for *tet*K and one for *tet*M. Of the three Er-resistant isolates, one had both *erm*B and *erm*C, one only *erm*C, and the other, *erm*B (Table 5).

**Table 4.** Antimicrobial susceptibility results, resistance genes detected and SCC*mec* typing of the 17 ovine and 28 human *S. haemolyticus* isolates

Source	Antimicrobial resistance^a^	Resistance genes^b^	SCC*mec* type C*mec* type
*Ovine isolates*			
Milk	Te	*tet*K	–
*Human isolates*			
			
Peritoneal fluid	–	–	–
Ear swab	–	*qac*A/B	–
Seminal fluid	Pn	*bla*Z	–
Seminal fluid	Pn-Er-Te	*bla*Z, *erm*C, *tet*K, *qac*A/B	–
Pus	Pn-Er-Km-Te	*bla*Z, *msr*A, *tet*K, *qac*A/B	–
Nasal swab	Pn-Er-Km-Te	*bla*Z, *erm*C, *tet*K, *smr*	–
Urine	Pn-Km-Te-Ox	*bla*Z, *tet*K, *smr*	Type IV
Nasal swab	Pn-Km-Trs-Ox	*bla*Z	Type V
Peritoneal swab	Er-Gn-Km-Te-Trs	*erm*C, *tet*K, *smr*	–
Blood	Pn-Er-Km-Te-Trs	*bla*Z, *erm*C, *tet*K, *smr*	–
Seminal fluid	Pn-Gn-Km-Te-Ox	*bla*Z, *tet*K, *qacA*/*B*, *smr*	Type IV
B.L. fluid^c^	Pn-Er-Gn-Km-St-Te	*bla*Z, *msr*A, *tet*K, *qac*A/B	–
Blood	Pn-Kf-Er-Gn-Km-Te	*bla*Z, *erm*C, *msr*A, *tet*K, *smr*	–
Ear swab	Pn-Er-Gn-Km-Trs-Ox	*bla*Z, *msr*A	Type V
Blood	Pn-Kf-Er-St-Amc-Ox	*bla*Z, *ermA*, *msrA*	Type IV
Seminal fluid	Pn-Er-Gn-Km-Te-Amc-Ox	*bla*Z, *erm*C, *tet*K, *qac*A/B	Type V
Nasal swab	Pn-Kf-Er-Gn-Km-Te-Trs-Ox	*bla*Z, *erm*C, *msr*A, *tet*K,	type V
Skin swab	Pn-Kf-Er-Gn-Km-Te-Amc-Ox	*bla*Z, *msr*A, *tet*K, *qac*A/B	NT
Nasal swab	Pn-Kf-Er-Gn-Km-Te-Amc-Ox	*bla*Z, *msr*A, *tet*K, *qac*A/B	NT
Oral swab	Pn-Kf-Er-Gn-Km-Te-Amc-Ox	*bla*Z, *msr*A, *tet*K, *qac*A/B	NT
Bile	Pn-Kf-Er-Gn- Km-St-Amc-Ox	*bla*Z, *erm*C, *msr*A, *qac*A/B	NT
Skin swab	Pn-Kf-Er-Gn- Km-St-Amc-Ox	*bla*Z, *erm*C, *msr*A, *qac*A/B, *smr*	NT
Nasal swab	Pn-Kf-Er-Gn-Km-Amc-Trs-Ox	*bla*Z, *msr*A, *qac*A/B	NT
Nasal swab	Pn-Kf-Er-Gn-Km-Amc-Trs-Ox	*bla*Z, *erm*C, *msr*A, *qac*A/B	Type V
Glans swab	Pn-Kf-Er-Gn-Km-Amc-Trs-Ox	*bla*Z, *erm*C, *msr*A, *qac*A/B	Type VII
Blood	Pn-Kf-Er-Gn-Km-St-Amc-Trs-Ox	*bla*Z, *erm*C, *msr*A, *qac*A/B, *smr*	NT
Peritoneal fluid	Pn-Kf-Er-Gn-Km-St-Amc-Trs-Ox	*bla*Z, *erm*C, *msr*A, *smr*	NT
Urine	Pn-Kf-Er-Gn-Km-St-Te-Amc-Trs-Ox	*bla*Z, *msr*A, *tet*K	Type V

a Antibiotic abbreviations: Pn, penicillin; Er, erythromycin; Te, tetracycline; Km, kanamycin; Ox, oxacillin, Trs, trimethoprim-sulphamethoxazole; Gn, gentamicin; St, streptomycin, Kf, cephalothin; Amc, amoxicillin-clavulanic acid.

b Resistance genes for Pn (*bla*Z), Er (*erm*A, *erm*B, *erm*C, *msr*A), Te (*tet*K, *tet*O, *tet*L, *tet*M), antiseptic agents (*qacA*/*B*, *smr*).

NT, isolate non typeable for SCC*mec*.

c Specimen abbreviations**:** B.L. fluid, bronchoalveolar lavage fluid.

**Table 5.** Antimicrobial susceptibility results, resistance genes detected and SCC*mec* typing of *S. chromogenes*, *S. simulans*, *S. warneri* and *S. caprae* collected from milk and of 10 minor human NAS isolates

Source				
*Ovine isolates*	Species	Antimicrobial resistance^a^	Resistance genes^b^	SCC*mec* type C*mec* type
Milk	*S. chromogenes* (*n* = 3)	Te	*tet*K	–
	*S. chromogenes* (*n* = 6)	Pn	*bla*Z	–
	*S. chromogenes*	Er	*erm*B	–
	*S. chromogenes*	Er	*erm*B, *erm*C	–
	*S. chromogenes*	Er-St	*erm*C	–
	*S. chromogenes*	Pn-Te	*bla*Z, *tet*M	–
	*S. chromogenes*	Pn-Te	*bla*Z, *tet*k	–
	*S. chromogenes*	St-Te	*tet*K	–
	*S. simulans*	Er	*erm*C	–
	*S. simulans*	Te	*tet*M	–
	*S. simulans*	Te	*tet*K	–
	*S. simulans*	St	–	–
	*S. caprae*	Pn	*bla*Z	–
	*S. caprae* (*n* = 2)	Pn-Te	*bla*Z, *tet*K	–
	*S. warneri*	Pn	*bla*Z	–
*Human isolates*				
Pus	*S. pasteuri*	Er	*erm*C, *msr*A	–
N.P. aspirate^c^	*S. capitis*	Pn	*bla*Z	–
Oral swab	*S. saprophyticus*	Te	*tet*K	–
Ear swab	*S. warneri*	–	*qac*A/B	–
Biopsy	*S. warneri*	Pn-Amc	*bla*Z,	–
Prosthesis	*S. hominis*	Pn-Te	*bla*Z, *tet*K	–
Blood	*S. hominis*	Pn-Er-Km-Te-Trs	*bla*Z, *erm*C *tet*K, *qac*A/B	–
Pleural fluid	*S. hominis*	Pn-Er-Gn-Km-Te-Trs	*bla*Z, *erm*C, *tet*K, *qac*A/B	–
Urine	*S. hominis*	Pn-Er-Gn-Km-Te-Trs-Ox	*bla*Z, *erm*C *tet*K	Type I

a Antibiotic abbreviations: Pn, penicillin; Er, erythromycin; Te, tetracycline; Km, kanamycin; Ox, oxacillin, Trs, trimethoprim-sulphamethoxazole; Gn, gentamicin; Amc, amoxicillin-clavulanic acid; St, streptomycin.

b Resistance genes for Pn (*bla*Z), Er (*erm*A, *erm*B, *erm*C, *msr*A), Te (*tet*K, *tet*O, *tet*L, *tet*M), antiseptic agents (*qacA*/*B*, *smr*).

c Specimen abbreviations: N.P. aspirate, nasopharyngeal aspirate.

Regarding the remaining ovine isolates, two of four *S. simulans* were resistant to Te (*tet*K or *tet*M gene), one to Er (*erm*C gene) and one to St. Two of the three *S. caprae* were resistant to both Pn and Te (*bla*Z and *tet*K genes) and one to Pn alone (*bla*Z). The single Pn-resistant isolate of *S. warneri* contained the *bla*Z gene (Table 5).

### Human NAS

Sixty-four NAS isolates of human origin were identified as: *S. haemolyticus* (*n* = 28), *S. epidermidis* (*n* = 26), *S. hominis* (*n* = 4), *S. capitis* (*n* = 3), *S. warneri* (*n* = 2) and *S. xylosus* (*n* = 1). Six additional isolates were identified by DNA sequencing as *S. lugdunensis* (*n* = 4), *S. pasteuri* (*n* = 1) and *S. saprophyticus* subsp. *bovis* (*n* = 1) (Table 1). Only 10 (14.3%) of the 70 isolates of NAS were susceptible to all antimicrobials tested, with the following distribution: *S. lugdunensis* (*n* = 4), *S. capitis* (*n* = 2), *S. haemolyticus* (*n* = 2) and one isolate each of *S. epidermidis* and *S. warneri*. The remainder were resistant to one (21.6%), two (13.4%) and three or more (65%) classes of antibiotics.

#### S. haemolyticus

All, but two, of 28 isolates of this species were resistant to one or more antimicrobials, with high rates to Pn (*n* = 25, 89.3%), Km (*n* = 23, 82.1%), Er (*n* = 22, 78.6%), Ox/Kx (*n* = 18, 64.3%) and Te (*n* = 15, 53.6%; the great majority being MDR) (Table 4). All 18 Ox-resistant *S. haemolyticus* isolates were *mec*A positive with SCC*mec* type V represented by six and SSC*mec* type IV in three. A single isolate of SCC*mec* type VII with recombinase genes *ccr*A5 and *ccr*B5 was also PCR-positive for the *ccrAB*_SHP_ allotype. Eight isolates were non-typeable for SCC*mec* (Table 4). All Pn-resistant isolates harboured the *bla*Z gene. Among the 22 Er-resistant isolates, five possessed only the *erm*C gene, eight only the *msr*A gene, eight carried simultaneously *erm*C and *msr*A genes, and one was positive for both *erm*A and *msr*A genes (Table 4). All 15 Te-resistant isolates had the *tet*K gene. Antiseptic-resistance genes: *qacA*/*B* alone, or *smr* alone, were detected in 42.8% (*n* = 12) and in 21.4% (*n* = 6) of the *S. haemolyticus* isolates, respectively. An additional three isolates possessed both *qacA*/*B* and *smr* genes.

#### S. epidermidis

All, but one, of the 26 *S. epidermidis* isolates were resistant to ⩾1 antibiotic (Table 2) with 21 (80.7%) resistant to Pn, 15 (57.7%) each to the macrolide Er and aminoglycosides, 14 (53.8%) to Ox/Kx and 4 (15.4%) to Te. Half of the species isolates were MDR. The 14 Ox/Kx-resistant isolates (SCC*mec* type II = 1, SCC*mec* type IV = 4 and SCC*mec* type III = 5). Four isolates were non-typeable for SCC*mec* and were also PCR-negative for *ccrAB5* and *ccrAB*_SHP_. All Pn-resistant isolates were positive for the *bla*Z gene; six of 15 Er-resistant isolates were positive only for the *msr*A gene, five the *erm*C gene and four both *erm*C and *msr*A genes. The four Te-resistant isolates carried the *tet*K gene, one of them also had the *tet*L gene. Nine (34.6%) isolates possessed the *qacA*/*B* gene alone and the same number only the *smr* gene. Three isolates from blood, skin swab and seminal fluid carried both genes. Interestingly, the *smr* gene was detected in the antimicrobial-susceptible *S. epidermidis* isolated from pus.

MLST analysis showed that ST59 and ST5 were each represented by six isolates and ST80 by five. ST981 proved to be a novel type.

#### Minor human NAS

Three of the four *S. hominis* isolates were MDR and one of them, isolated from urine, was including Er (*erm*C gene) and Te (*tet*K gene); one of them, isolated from urine, was SCC*mec* type I. Of note, each of the four *S. hominis* isolates was positive for the *bla*Z gene and two also harboured the *qac*A/B gene (Table 5). *S. pasteuri* and *S. capitis* isolates were resistant to Er (both *erm*C and *msr*A genes) and Pn (*bla*Z gene), respectively. *S. saprophyticus* subsp. *bovis*, resistant to Te, possessed the *tet*K gene. In the *S. warneri* isolate, collected from an ear swab and susceptible to all antibiotics tested, the *qac*A/B gene was found (Table 5).

## Discussion

This study is the result of an integrated collaboration between veterinary and human health care professionals to determine whether NAS recovered from human infections share some genetic characteristics with those circulating in sheep. In the current study, 125 NAS from ovine mastitis and 70 from human clinical specimens were assigned to species level by PCR-RFLP of the *gap* gene. Among the ovine isolates, *S. epidermidis* was the most common followed by *S. chromogenes* and *S. haemolyticus*, while among human isolates *S. haemolyticus* and *S. epidermidis* were predominant, followed by *S. lugdunensis* and *S. hominis*.

*S. epidermidis* is widely recognised to be the most prevalent NAS recovered from ovine mastitis and human clinical specimens [1, 7, 29]. This study confirms our previous findings that ovine isolates of this species is the major reservoir of antimicrobial resistance genes, in particular for tetracycline and penicillin [6, 7]. However, the frequency of MDR in sheep isolates was considerably lower than that for human isolates (1.6% *vs*. 58.6%). Among the latter group, MDR was associated more frequently with *S. haemolyticus* (35.7%) than *S. epidermidis* (18.6%). This finding is in agreement with previous reports of resistance to a wide range of antimicrobials among *S. haemolyticus* from human clinical specimens, and also supports the view that this species may constitute an important reservoir for the transfer of resistance genes to other *Staphylococcus* species [30, 31].

None of the ovine NAS was methicillin resistant in contrast to 53.8% of human isolates. The *mec*A gene is a constituent of the mobile genetic element SCC*mec*, which acts as a vehicle for horizontal transfer of antibiotic resistance genes [32]. Among the oxacillin-resistant *S. epidermidis*, we found type III to be the predominant SCC*mec*, followed by type IV, the latter being common in human isolates of the species [32, 33], while SCC*mec* type III is more widely distributed among NAS species; the mechanism responsible for this is not understood. The identification of SSC*mec* type V as the most prevalent in *S. haemolyticus* is consistent with previous reports of the high frequency of class C2 *mec*-*ccr*C complexes (type V) in *S. haemolyticus* isolated from outpatients living in Algeria, Mali, Moldova, Cambodia and China [32, 33]. By contrast, *S. haemolyticus* collected in South Brazil and India mainly harboured SCC*mec* type I [34, 35]. We identified *ccr*A5–*ccr*B5 recombinase genes in one of the *S. haemolyticus* isolates from a human glans specimen. This gene complex has only been reported in *S. pseudintermedius* from animals [21], and to the best of our knowledge, this is the first report describing the detection of SCC*mec* type VII in *S. haemolyticus* from human clinical specimens. The *ccrAB*_SHP_ allotype was described by Pi *et al*. [22] in *S. haemolyticus* isolates resistant to methicillin and harbouring arginine catabolic mobile element (ACME) cluster genes. This group suggested that *ccrAB*_SHP_ had a similar function to the known *ccr* allotype, that is, to catalyse the integration and excision of SCC*mec* and ACME. Our inability to establish the SSCmec type for eight of the *S. haemolyticus* studied here could be attributed to the presence of novel *ccr* allotypes.

The plasmid-located *tet*K gene was the most prevalent determinant encoding tetracycline resistance, indicating that the resistance mechanism is mainly mediated by the tetracycline efflux pump [36]. In *S. aureus*, a strong association between *tet*K gene and SCC*mec* type V has been reported by Larsen *et al*. [37] but we did not observe any such correlation in our NAS isolates, although the *tet*k gene was amplified in oxacillin-negative isolates. The detection of *msr*A gene encoding an ATP-dependent efflux pump among the erythromycin-resistant NAS is consistent with other reports from France [38] and Tunisia [39].

The inappropriate use of disinfectants can lead to a concentration gradient in the environment, which could select for isolates with reduced susceptibility to these agents. Here, we found a substantial difference between animal and human NAS as QAC resistance was identified in only a single ovine isolate compared to 42 (60%) of 70 human isolates. The ovine *S. epidermidis* strain harboured the *smr* gene whereas in human isolates, *qacA*/*B* genes were more prevalent (37.1%) than the *smr* gene (25.7%); both genes were similarly distributed in *S. haemolyticus* and *S. epidermidis* of human origin (was 70% and 69%, respectively). The predominance of *qacA*/*B* genes compared to the *smr* gene was also observed in NAS from Hong Kong [40] and Turkey [13]. Furthermore, it is noteworthy that almost all MDR-*S. haemolyticus* isolates from nasal, skin and oral swabs of patients in intensive care unit were PCR-positive for *qacA*/*B* genes.

NAS may therefore play a clinically significant role as reservoirs of resistance genes, especially *S. epidermidis* in sheep and *S. haemolyticus* in humans. The carriage of such genes in human NAS and the prevalence of MDR isolates were markedly higher compared with those of ovine origin. Similarly, no methicillin-resistant strains were found in the latter group which may reflect a more appropriate use of antimicrobials in humans as compared with dairy ruminants. Moreover, these findings may suggest that human NAS represent reservoirs of resistance genes which are transmissible to sheep isolates especially if they constitute part of the commensal skin microbiota of animal care or farm workers. On the other hand, the role of sheep NAS as reservoirs of resistance genes for human NAS seems reasonably lower. Nevertheless, this will require further studies, as well as periodic surveillance measures to monitor the spread of antimicrobial genes and maintain control.

Concerning the presence of sequence types in the two species, MLST genotyping of *S. epidermidis* revealed that ST225, ST6 and ST100 predominated in sheep, compared with T59, ST5 and ST80 in humans. Reports of sequence typing studies on animal-derived *S. epidermidis* are limited and currently none has analysed ovine milk isolates. Among human *S. epidermidis*, ST2 has usually been reported to be the most prevalent in humans [16, 41], but only one representative isolate of this ST was identified in our collection. Similarly, to our knowledge, only two studies have so far reported ST5 to be more common than ST2 [42, 43], which might be indicative of a different distribution in Sardinia from other geographical areas. Finally, it is noteworthy that one *S. epidermidis* ovine isolate was typed as ST5, which might suggest a possible human to animal transmission. This highlights the risk for strain and gene passage in this direction and underscores the need for periodic surveillance measures.

In conclusion, we believe that this is the first study to investigate and compare the occurrence of antimicrobial/antiseptic resistance and associated genetic determinants in human and ovine NAS in Italy. Our results document the great importance of NAS as reservoirs of resistance genes, in particular *S. epidermidis* for sheep and *S. haemolyticus* for humans. As many of the resistance determinants are located on transmissible plasmids, we propose that periodic surveillance might provide important information relevant to the control of animal and human infections, and thus help to limit the spread of MDR bacteria from humans to animals.

## Data Availability

The authors confirm that the data supporting the findings of this study are available within the paper.
